# Neuroinflammation, Pericyte Dysfunction, and Alzheimer’s Disease-Associated Gene Expression and Pathway Activation in the Brain of SARS-CoV-2-Infected Mice

**DOI:** 10.3390/v18070783

**Published:** 2026-07-17

**Authors:** Akinkunmi O. Lawal, Ikechukwu B. Jacob, Vignesh Karnik, Hongkuan Fan, Saravanan Thangamani, Paul T. Massa, Guirong Wang

**Affiliations:** 1 Department of Surgery, SUNY Upstate Medical University, Syracuse, NY 13210, USA; lawala@upstate.edu (A.O.L.); ikechukwujacob86@gmail.com (I.B.J.); karnikv@upstate.edu (V.K.); 2 Department of Microbiology & Immunology, SUNY Upstate Medical University, Syracuse, NY 13210, USA; thangams@upstate.edu (S.T.); massap@upstate.edu (P.T.M.); 3 Department of Pathology and Laboratory Medicine, Medical University of South Carolina, Charleston, SC 29425, USA; fanhong@musc.edu; 4 Department of Neurology, SUNY Upstate Medical University, Syracuse, NY 13210, USA

**Keywords:** Alzheimer’s disease, AD-related gene expression, human ACE2 transgenic mice, neuroinflammation, pericyte dysfunction, SARS-CoV-2 infection

## Abstract

SARS-CoV-2 infection leads to extrapulmonary complications in multiple organs, including the brain, both in the short-term and long-term. The neurological manifestation of SARS-CoV-2 infection ranges from benign signs like loss of smell and loss of taste to severe complications like encephalitis, stroke, and exacerbation of Alzheimer’s disease (AD) progression. Pericytes are mural cells of the brain vasculature that help maintain the blood–brain barrier (BBB), regulate cerebral blood flow (CBF), modulate neuroinflammation, and clear toxic materials, including amyloid beta. Pericytes express ACE2, the receptor for SARS-CoV-2, and therefore may be targeted by either direct virus infection or virus-induced inflammatory cytokines induced by the virus in the brain. To further study the effects of SARS-CoV-2 on pericytes and BBB integrity, the long-term effects of SARS-CoV-2 infection on brain pericytes, inflammation, and other neuropathological complications were analyzed in mice. K18 (human ACE2 transgenic) mice were infected with 10^3^ PFU of SARS-CoV-2 (delta strain), and the brains were analyzed at 6, 14, and 30 days post-infection (dpi). A significant reduction in the weight of infected mice was observed by 6 dpi. Viral nucleocapsid protein and infectious SARS-CoV-2 were observed in the brains of all mice by 6 dpi, and in some mice by 14 dpi, but not at 30 dpi. This observation suggests viral neurotropism with subsequent clearance at later timepoints. Despite virus clearance, the levels of inflammatory mediators, including TNF-α and IFN-γ were significantly elevated up to 30 dpi. We also observed a significant reduction in the level of brain pericytes by 14 dpi up to 30 dpi. Importantly, an increase was observed in the level of Friend Leukemia Integration 1 (FLI-1), a transcription factor known to promote pericyte cell death, from 14 dpi up to 30 dpi. The level of amyloid beta 1–42 was elevated in the brain of infected mice at 6 dpi, and this was maintained up to 30 dpi, and there was a decrease in neuronal density from 14 to 30 dpi. Furthermore, we observed an increased expression of Alzheimer’s disease (AD)-associated genes like PSEN1, BACE1, and APP. Furthermore, there was increased activation of several neurodegenerative pathways, including “G alpha (z) signaling pathway”, “Apelin muscle signaling pathway”, and “G beta-gamma (Gβγ) signaling”, in the brains of infected mice compared to control mice. Collectively, the observed neuropathology and unique molecular markers of neurodegenerative disease progression provide a novel mechanism by which COVID-19 may promote dementia/AD by contributing to pericyte loss and BBB dysfunction during infection.

## 1. Introduction

Since the emergence of the SARS-CoV-2 infection in late 2019, there have been approximately 800 million reported cases, with over 7 million confirmed COVID-19 deaths globally, making the disease a significant threat to public health [1]. SARS-CoV-2 is primarily a respiratory disease and induces lung inflammation, resulting in acute respiratory distress syndrome (ARDS) and death in severe cases [2,3,4]. Mortality aside, there are reports of extrapulmonary complications, including neurological dysfunctions, during and post COVID-19 [5,6]. SARS-CoV-2 infection affects multiple organ systems including the circulatory, digestive and central nervous systems, thereby potentiating a wide range of systemic disorders [7]. This is unsurprising because the virus spike protein binds to the human angiotensin-converting enzyme (hACE2) to initiate entry into host cells, and hACE2 is widely expressed in multiple organs, including the brain [8,9]. The virus also uses other host proteins, such as neuropilin and vimentin, which are present in the brain, for cellular entry [10,11]. COVID-19 patients experience neurological complications ranging from benign symptoms like loss of smell and taste to severe complications such as delirium, loss of memory, encephalitis, and seizures [12]. In addition, SARS-CoV-2 infection induces BBB breakdown and cerebral blood flow (CBF) constriction, as observed in postmortem tissues of COVID-19 patients, and studies suggest that BBB breakdown plays a significant role in the development of the neurological complications observed after SARS-CoV-2 infection [13,14,15]. It has been established that BBB disruption drives multiple pathways of neurodegeneration, ranging from brain fog to Alzheimer’s disease [16,17]. Interestingly, studies show that there is an increased risk of Alzheimer’s disease (AD) months after recovery from acute COVID-19, especially in older adults [18,19,20]. Furthermore, accelerated progression of cognitive decline was observed in patients with pre-existing dementia after SARS-CoV-2 infection [21,22]. However, the cellular and molecular mechanisms underlying BBB breakdown in COVID-19 patients are not fully understood. Amruta et al. showed that SARS-CoV-2 infection, using a mouse-adapted variant, induces neuroinflammation and a reduction in claudin–5 level, suggesting BBB disruption [23]. In addition, other studies have shown neurological complications such as microgliosis, astrocyte activation, perivascular lymphocyte cuffing, and neuronal death post SARS-CoV-2 infection in mice [24,25,26]. Given the crucial role pericytes play in the maintenance of the BBB and CBF, it is important to investigate the effect of SARS-CoV-2 infection on pericytes.

Brain pericytes form the neurovascular unit together with neurons, astrocytes, and endothelial cells. They perform various functions like blood–brain barrier maintenance, inflammatory cell recruitment, cerebral blood flow control, and neuroinflammation mediation [27,28]. Viruses like Zika and Japanese encephalitis virus (JEV) that are known to induce neurological disorders infect pericytes and lead to pericyte death or dysfunction [29,30]. Human brain pericytes express ACE2, the primary receptor for SARS-CoV-2, thereby making these cells uniquely susceptible to infection and loss [31,32]. Taken together, SARS-CoV-2 may have significant effects on pericyte function in the BBB in ways that drive AD progression.

Previous studies demonstrate that SARS-CoV-2 infects pericytes in various organs, including the lungs, heart, and brain, leading to complications in these organs [31,33,34,35]. Brain pericytes maintain the integrity of the BBB by inducing the polarization of astrocyte end feet that wrap around blood vessels and by regulating the expression of BBB tight junction proteins in the brain [14,27]. In addition, neurons secrete neurotransmitters such as glutamate and noradrenaline, which induce pericyte relaxation and contraction around capillaries, thereby controlling cerebral blood flow [36,37]. Importantly, pericyte loss or dysfunction results in BBB breakdown, CBF constriction, and neuroinflammation, thereby inducing a wide range of neurological disorders [38,39,40].

Alzheimer’s disease is a chronic neurological disease characterized by the presence of positive and negative lesions in the brain. The positive lesions include amyloid plaques, tau-containing neurofibrillary tangles, and glial activation, while the negative lesions comprise synaptic damage, neuronal loss, and general brain atrophy [41,42,43]. Longitudinal studies show gradual amyloid beta deposition in the memory region of the brain and tau pathology 10 to 20 years before the clinical manifestation of AD [44,45,46,47,48], followed by cerebral hypometabolism and hippocampal dystrophy. AD causes significant and gradual neurological deficits and finally results in death within 5 to 12 years following symptom onset [46,47,49]. The amyloid cascade hypothesis is the most widely accepted AD pathogenesis model since it was postulated by Hardy and Higgins in 1992. They postulated that Aβ deposition induces AD pathology by initiating the formation of neurofibrillary tau tangles, vascular damage, synaptic loss, and the ensuing cognitive decline [47,50]. Amyloid precursor protein (APP) is rapidly produced by neurons and can be proteolyzed through various pathways, which may result in the production of Aβ [51]. In the pathogenic, amyloidogenic pathway, beta-secretase (BACE1) and gamma-secretase sequentially cleave APP to produce Aβ peptides, which are released into the extracellular space, and the APP intracellular domain (AICD) that is released into the intracellular space. Gamma (γ)-secretase comprises four proteins, two of which are presenilin 1 and presenilin 2 (PSEN1 and PSEN2), which form the catalytic core, and gain-of-function mutations in the PSEN1 and PSEN2 genes potentiate AD development [52,53,54]. Tau, another hallmark of Alzheimer’s disease, is a microtubule-associated protein encoded by the MAPT (microtubule-associated protein tau) gene on chromosome 17 [55]. The protein is alternatively spliced to produce a total of six distinct three-repeat (3R) and four-repeat (4R) isoforms due to alternative splicing of exons 2,3 and 10 [56,57,58]. Several studies show an overexpression of 4R tau in vulnerable regions of the brain and neurons bearing hyperphosphorylated tau tangles in the brains of AD patients. However, that notion is still being debated as neurofibrillary tau tangles in the brain comprise both the 3R and the 4R isoforms [59,60,61,62,63]. MAPT overexpression and tau accumulation induce tauopathies and other neuropathologies, even if the predominant form is the 3R isotype [64].

In this study, we present evidence that SARS-CoV-2 infection induces pericyte death and severe neuroinflammation in K18 mice. These neuropathological features persist for an extended period of up to 30 dpi. The infected mice also showed significantly elevated Aβ1–42 levels, fibrin deposition, and a significant decrease in claudin 5 levels in the brains of infected mice by 14 dpi, suggesting BBB disruption. Furthermore, we observed a significant decrease in NeuN expression in the brains of infected mice by 14 dpi, increased expression of multiple AD-related genes, and activation of neurodegenerative pathways in the brains of infected mice compared to mock-infected animals. Lastly, IPA disease machine learning predicted neurodegenerative diseases using the upregulated genes acquired from the RT^2^ Profiler^TM^ PCR Array analysis. Taken together, these data indicate that SARS-CoV-2 infection induces neuroinflammation, neuronal death, BBB disruption, and may potentiate the development of AD and other Alzheimer’s disease-related dementias (ADRD). The data presented support previous epidemiological studies that show an increased risk of developing neurological complications post COVID-19.

## 2. Materials and Methods

### 2.1. Mice

SARS-CoV-2-susceptible hACE2-transgenic K18 mice (K18 mice) were used for this study. The initial K18 mouse stock was obtained from Jackson Lab (Bar Harbor, ME, USA). The mice were bred and maintained at the SUNY Upstate Animal Core Facility in a temperature-controlled, pathogen-free housing at 22 °C. The mice were genotyped for the hACE2 gene as described by Ikechukwu et al. [65], and hACE2-positive mice were used for the study. For this study, both male and female mice, aged 12 to 16 weeks, were used. Experiments were approved by the SUNY Upstate Medical University Institutional Animal Care and Use Committee. The mice were transported into the infection room at least 24 h before infection. A total of 98 mice (16 mock-infected and 92 infected) were used for this study based on the survival rate post-infection. Mice were randomly assigned to the control or the infected group based on the last two digits of their cage identification, and each cage had a minimum of 2 mice and a maximum of 5. Mock-infected mice and infected mice were assigned before the experiments. The surviving infected mice were randomly assigned to 6 dpi, 14 dpi, and 30 dpi during the experiments.

### 2.2. Mouse Infection and Sample Collection

We used a low-passage infectious SARS-CoV-2 (B.1.617.2, Delta variant) for this study. This virus strain has been used for previous laboratory studies and has been confirmed to successfully infect K18 mice [65,66]. SARS-CoV-2 and mock-infected mice were anesthetized with isoflurane and intranasally infected with 1 × 10^3^ pfu of SARS-CoV-2 (in 30 μL MEM; 15 μL per nostril) and 30 μL MEM, respectively. The mice were weighed daily, and a 20% decrease in weight was used as the endpoint. The mice were euthanized at 6, 14, and 30 days post-infection by exsanguination under anesthesia, and the brain tissues were collected (*n* = 6–10 mice per group) and immediately transferred to −80 °C. Some mice were dissected along the abdomen, and the whole mice were fixed by heart perfusion with 10% neutral buffered formalin (NBF), and then stored in 10% NBF for histological and immunological analyses (*n* = 6–8 mice per group).

### 2.3. Histopathological Analysis

The previously fixed brains were paraffin-embedded, and 5 µm sections of each tissue sample were cut, stained with Hematoxylin and Eosin (H&E) stains, and assessed under a microscope for the influx of inflammatory cells into the brain as previously described [67].

### 2.4. Immunohistochemical Analysis

The fixed tissues were embedded in paraffin and then sectioned at 5 µm thickness onto slides for further processing. Sections were dewaxed for 30 min at 60 °C, deparaffinized by washing in xylene twice, and rehydrated with decreasing concentrations of ethanol from 100% to 50%. Sections were then rinsed 2 times in deionized water and boiled in 10 mM citrate (pH 6.0) for 10 min and cooled for 30 min at room temperature for antigen retrieval. Background staining was blocked with 10% BSA in PBS for 1 h at room temperature before incubating the slides at 4 °C overnight with rabbit anti-SARS-CoV-2 NP antibody (cell signaling, Danvers, MA, USA, #33336S; 1:100), rabbit anti-CD13 antibody (cell signaling, MA, USA, #D6V1W), rabbit anti-beta amyloid antibody (ABCAM, Fremont, CA, USA, #ab201060; 1:1000), rabbit anti-fibrinogen antibody (ABCAM, CA, USA, #ab92572; 1:100) or anti-NeuN antibody (ABCAM, CA, USA, #ab177487; 1:200). The following day, the slides were washed 3 times in PBS and incubated with biotinylated goat anti-rabbit antibody for 1 h, followed by ABC/HRP complex (peroxidase rabbit IgG PK-4001; Vector Laboratories, Newark, CA, USA). Slides were then stained with 3′3-diaminobenzidine (DAB) (SK-4100; Vector Laboratories, CA, USA) for 5–10 min and counterstained with hematoxylin QS counterstain (Vector Laboratories, CA, USA, H-3404-100). Images were acquired using the Nikon Eclipse TE2000-U microscope (Nikon Corporation, Tokyo, Japan) and analyzed using ImageJ software (Version 1.45S). For Immunofluorescence, the slides were incubated with the appropriate fluorescent-tagged (Alexa 488 and Alexa 594) secondary antibody for 1 h at room temperature. Afterwards, excess primary antibody was washed off with PBST thrice. Every wash was for 5 min unless otherwise stated.

### 2.5. Western Blot Analysis

Brain tissues from each group were homogenized in protease and phosphatase inhibitors (Roche, Indianapolis, IN, USA)-containing RIPA buffer. BCA^TM^ protein analysis (ThermoFisher Scientific, Rockford, IL, USA; #23235) was done on the homogenates following the manufacturer’s protocol to determine the protein concentration in the homogenates. 20 µg of total protein was resolved by SDS-PAGE on a 10% gel and transferred onto PVDF membranes (Bio-Rad). The blots were blocked with 5% non-fat milk in TBST for 1 h and incubated overnight at 4 °C with anti-FLI1 (ThermoFisher, IL, USA, #MA1-196; 1:500), CD13, Claudin 5 (ThermoFisher, IL, USA, #35-2500; 1:1000), or amyloid precursor protein (ABCAM, CA, USA, #ab32136; 1:10,000) antibodies. As loading control, blots were stripped and re-probed with the GAPDH or beta-ACTIN antibody. Subsequently, the membranes were incubated with goat anti-mouse or anti-rabbit IgG HRP-conjugated secondary antibody (Bio-Rad, Hercules, CA, USA; #1620177) and developed using ECL Western Blotting Substrate (ThermoFisher Scientific, IL, USA). The expression of proteins was quantified using an imaging system (Bio-Rad Chemi-Doc XRS+ imaging system, Bio-Rad, CA, USA). Each analysis was replicated 3–4 times.

### 2.6. Plaque Assay

To quantify infectious titers in the brain at different time points by plaque assay, about 10^5^ Vero cells are seeded/well in 24-well plates and incubated at 37 °C overnight. The near confluent monolayers of Vero E6 are then infected with 100 μL of 10-fold serial dilutions of homogenates from each group. The cells are incubated for 1 h with intermittent rocking at 15 min intervals to prevent drying out. The homogenates are removed, and the cells are overlaid with 2% methylcellulose and incubated for 3 days. Cells are then fixed with 10% formalin for 1 h, stained with 0.05% (*w*/*v*) crystal violet, and plaques are counted. Each assay was replicated twice.

### 2.7. Transcriptomics Analyses

We examined the expression of Alzheimer’s disease-associated genes in the brain of SARS-CoV-2 infected mice using the RT^2^ Profiler^TM^ PCR Array for Mouse kit (Qiagen, Boston, MA, USA, Cat. No.: 330231; GeneGlobe ID: PAMM-057Z). Total RNA was isolated from the homogenized brain tissues using the RNA extraction miniprep kit (Zymo Research, Irvine, CA, USA, #R1055) according to the manufacturer’s protocol. The extracted RNA’s concentrations were then determined using the NanoDrop Spectrophotometer (Thermo Scientific, IL, USA), and 1 µg of the total RNA of each sample was reverse transcribed using the iSCRIPT Reverse Transcription kit (Bio-Rad, CA, USA, #1708841) according to the manufacturer’s protocol. The Applied Biosystem StepOnePlus qPCR machine (ThermoFisher, IL, USA) was used for the quantification of the 96 genes in each sample using the RT^2^ Profiler^TM^ PCR plates. The raw data were exported to Excel spreadsheets, combined, and analyzed using the Qiagen PCR analysis webpage (GeneGlobe Data Analysis Center). The mRNA expression of each gene in each sample at the different timepoints was normalized to the housekeeping genes (HSP90, Beta Actin, B2M, and Gusb). Fold change values were compared between the mock and each time point, and between time points. Gene expression fold change is considered significant when fold change is >2.0 (*p*-value < 0.05). In addition, we validated the expression of selected differentially expressed genes by RT-qPCR using the following primers. BACE1 (F: TGCTGCCATCACTGAATCGGAC; R: GGAATGTGGGTCTGCTTCACCA), PSEN1 (F: GAGACTGGAACACAACCATAGCC; R: AGAACACGAGCCCGAAGGTGAT), PSEN2: (F: GCTGTTTGTGCCTGTCACTCTG; R: TGTGTCCTCAGTGAATGGCGTG), MAPT (F: CCTGAGCAAAGTGACCTCCAAG; R: CAAGGAGCCAATCTTCGACTGG) [68]. RNA samples were reverse transcribed by priming for 5 min at 25 °C, reverse transcription at 46 °C for 20 min, and RT inactivation at 95 °C for 1 min. The cDNA was amplified by denaturation/polymerase activation for 30 s at 95 °C followed by 40 cycles of denaturation (95 °C for 10 s) and extension (60 °C for 60 s).

### 2.8. Signaling Pathway Analysis

Signaling pathway analysis was done with the Ingenuity Pathway Analysis software from Qiagen (QIAGEN, Redwood City, CA, USA) using the core analysis tab. Core analysis was done using the data generated from the RT^2^ Profiler^TM^ PCR Array analysis, and the results generated from the IPA include ‘top canonical pathways’, “diseases and functions”, and “gene networks”. Activated canonical pathways are ranked by the ratio of the number of upregulated genes in the imputed data to genes that are mapped into specific pathways in the IPA database.

### 2.9. ELISA Assay

Levels of TNF-α (Lot# 227846-003), IL-1β (#372944-002), IL-6 (#330384-008), and IFN-γ (#300277-008) (ThermoFisher Scientific, IL, USA) in brain homogenates were measured by enzyme-linked immunosorbent assay (ELISA). All measurements were made in duplicates, and experiments were repeated and performed according to the manufacturer’s provided protocol.

### 2.10. Statistical Analysis

All experimental data are presented as mean ± standard error and statistically analyzed using GraphPad Prism 8.0 (GraphPad Software, San Diego, CA, USA). Comparisons among groups were conducted using one-way ANOVA or *t*-test. Results were considered statistically significant when *p* < 0.05. Data were analyzed for outliers before proceeding with one-way ANOVA.

## 3. Results

### 3.1. SARS-CoV-2 Exhibits Neurotropism and Induces an Influx/Expansion of Inflammatory Cells into the Brain

**Figure 1:**
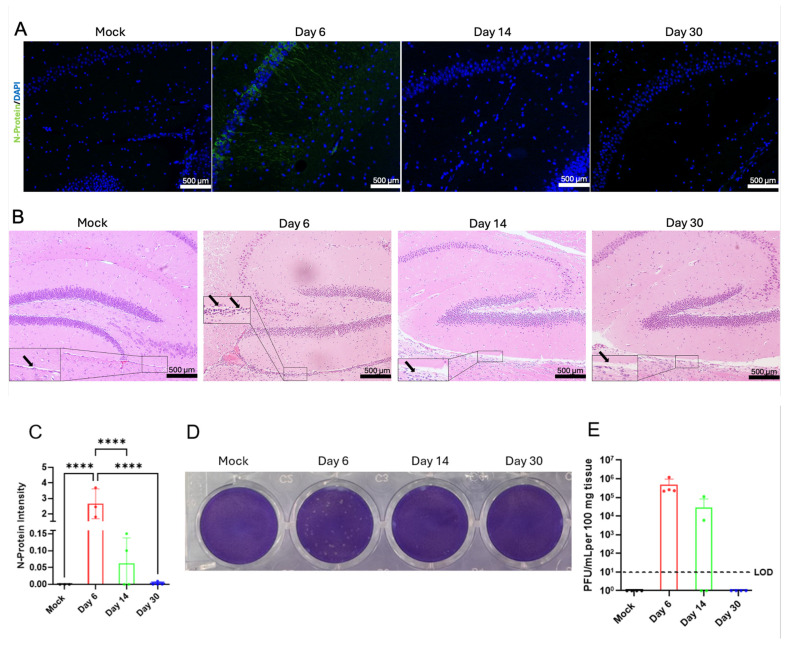
Presence of SARS-CoV-2 particles and immune cell influx/expansion in the brain of K18 mice post infection. (**A**): Immunofluorescence assay for SARS-CoV-2 nucleocapsid (*n*) protein following intranasal inoculation of SARS-CoV-2 in K18 mice shows the presence of the viral N-protein (green) in the brain tissue of all mice at 6 dpi but not at 30 dpi or mock-infected. SARS-CoV-2 N-protein was detected in 50% of the mice in reduced quantities at 14 dpi (Magnification: 200×). (**B**): H&E staining of brain sections (hippocampus area) from SARS-CoV-2 and mock-challenged mice showing increased level of meningeal accumulation of inflammatory cells at 6 dpi but reduced levels at 14 dpi and 30 dpi. Arrows show presence of perivascular inflammation (Magnification: 200×). (**C**): Quantification of N-protein between the different groups. *n* = 3–4 mice per group (Statistical Analysis: one-way ANOVA). (**D**): Plaque assay with brain tissues of SARS-CoV-2-infected mice. Infectious SARS-CoV-2 virus was detected in the brain tissue of all 6 dpi mice but not at 30 dpi or in mock-infected. SARS-CoV-2 was detected in 50% of the mice in reduced quantities (10× to 100× less) at 14 dpi. (**E**): Quantification of infectious virus in brain homogenates of the different groups presented in Log_10_. *n* = 4 mice per group (**** *p* < 0.0001 reflects the level of statistical significance between groups).

SARS-CoV-2 RNA has been detected in the brains of postmortem COVID-19 patients [69,70], and there is persistence of SARS-CoV-2 spike protein in the skull-meninges-brain axis of COVID-19 patients post-acute infection [71]. In addition, studies show that SARS-CoV-2 can infect different brain cells [72,73], and there is an increase in the expression of ACE2 in the brain of COVID-19 patients [74,75]. To examine whether SARS-CoV-2 infects the brain, we performed immunofluorescence analysis for SARS-CoV-2 N-protein in the brain of mock-infected mice and infected mice at 6, 14, and 30 dpi (Figure 1A,C). We observed a high level of N-protein staining by 6 dpi, whereas no N-protein was detected in the brains of 30 dpi mice. For 14 dpi, we observed low levels of N-protein staining in two of the four mice analyzed. Histological analysis of the brain sections also showed a moderate influx/expansion of inflammatory cells around the hippocampus by 6 dpi (Figure 1B). N protein staining of pericytes was not observed at any stage of infection, suggesting a lack of infection of these cells (Supplementary Figure S2). Since the presence of viral protein does not confirm the presence of fully packaged, infectious SARS-CoV-2 particles, we performed plaque assays to confirm the presence of infectious virus in the brains of infected mice. Interestingly, the result of the analysis mirrors that of the N-protein analysis. There was a high level of infectious virus in the brain by 6 dpi, low levels of infectious virus in 50% of the mice observed at 14 dpi, and no infectious virus in the brain by 30 dpi (Figure 1B,D). These data show that SARS-CoV-2 exhibits neurotropism, induces histopathological changes in the brain, and infection appears to be localized primarily to hippocampal neurons and apparently not cells constituting the BBB, including pericytes.

### 3.2. SARS-CoV-2 Infection Induces Pericyte Death

**Figure 2:**
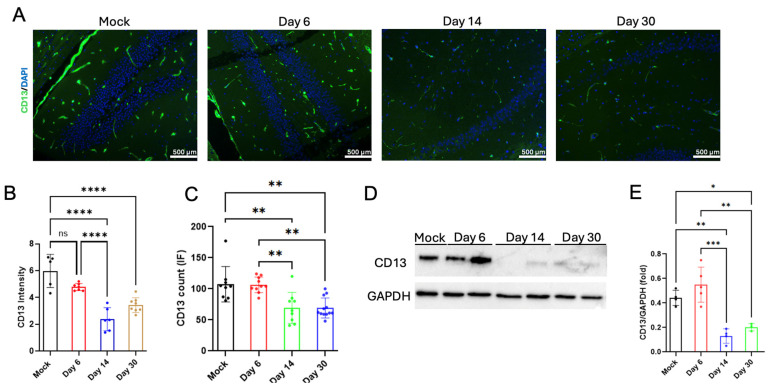

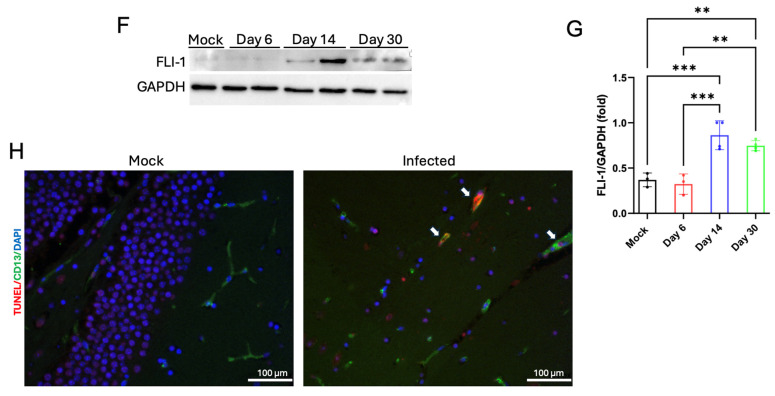
Decreased level of CD13 and increased level of FLI-1 in the brain tissues of SARS-CoV-2 infected mice compared to uninfected mice at 14 dpi and 30 dpi. (**A**): Immunofluorescence assay of CD13 in brain tissues of SARS-CoV-2 infected and mock mice. Infected mice show decreased levels of pericytes in the brain by 14 dpi and 30 dpi compared to uninfected mice (Magnification: 200×). (**B**): Quantification of CD13 immunofluorescence signals among the different groups shows a significant reduction in pericytes by 14 and 30 days compared to mock. No significant difference between 6 dpi and mock. *n* = 5–8 mice per group. (**C**): Analysis of CD13 counts per field in mock, 6 dpi, 14 dpi, and 30 dpi mice (9–12 fields per group). (**D**): Western blot of whole brain lysates shows a reduced level of CD13 in the brain at 14 dpi and 30 dpi. (**E**): Quantification of CD13 Western blot signals shows a significant reduction in the level of CD13 (Pericyte) in infected mice at 14 dpi and 30 dpi vs. mock-infected mice. No significant difference between 6 dpi and mock was observed. *n* = 3–5 mice per group. (**F**,**G**): Increased level of FLI-1 transcription factor in the whole brain of infected mice compared to uninfected. Significance is achieved by 14 dpi and 30 dpi compared to mock. *n* = 3–4 mice per group. (**H**): TUNEL assay + CD13 immunofluorescence assay. Arrows show co-localization of TUNEL and CD13 fluorescence signals shows pericyte death in the brain post-infection: (Magnification: 400×). Statistical Analysis: One-way ANOVA. The data are presented as mean ± SEM. (ns = no significance, * *p* < 0.05; ** *p* < 0.01; *** *p* < 0.001; **** *p* < 0.0001 reflect the levels of statistical significance between groups).

Due to the critical roles of pericytes in maintaining healthy brain homeostasis [38,39] and studies showing pericyte infection by SARS-CoV-2 in different organs [31,32,34], we assessed pericyte levels in the brains of infected compared to mock-infected mice. We stained for CD13, a widely used marker of pericytes, by immunofluorescence. Despite the lack of apparent pericyte infection in this model, there was a significant reduction in CD13 immunofluorescence in the hippocampal region of the brain by 14 dpi compared to 6 dpi and mock. This low level of CD13 was maintained until 30 dpi (Figure 2A,B). To confirm this result, we also probed CD13 in the whole brain by Western blot and observed similar results. The level of CD13 in the brain was significantly depleted by 14 dpi and 30 dpi compared to 6 dpi and mock (Figure 2D,E). Overall decrease in cell staining intensity in sections was confirmed by ImageJ software analysis. Importantly, when CD13-positive cells were counted at different dpi, the number of cells per unit area was significantly decreased at 14 dpi and 30 dpi, indicating loss of these cells (Figure 2C). To determine the possible cause for pericyte loss, we analyzed the expression of Friend Leukemia virus integration 1 (FLI1), which has been shown to play a key role in pericyte death and dysfunction in inflammatory disease models. As anticipated, we observed a significant increase in FLI-1 expression at 14 dpi and 30 dpi compared to 6 dpi and mock (Figure 2E,F). To analyze whether the pericytes were undergoing cell death, we double-stained cells for the TUNEL apoptotic marker and CD13 and observed a co-localization of CD13 with the TUNEL signal (Figure 2G). These data suggest that pericytes are being lost through the induction of apoptotic cell death pathways. Whether this occurs through direct viral infection or inflammation-induced cell death is presently unclear.

### 3.3. Inflammation Analysis in the Brain Post SARS-CoV-2 Infection

**Figure 3:**
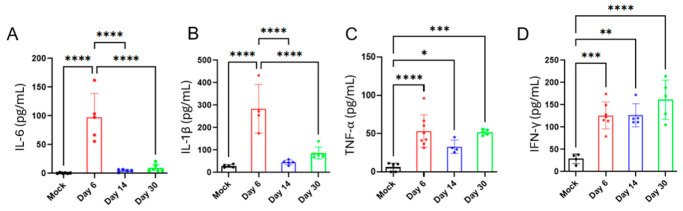
Chronically altered levels of pro-inflammatory cytokines in the brain of K18 mice post-infection. (**A**): Levels of IL-6 in whole brain lysates were measured using ELISA. (**B**): Levels of IL-1β in whole brain lysates were measured using ELISA. (**C**): Levels of TNF-α in whole brain lysates were measured using ELISA. (**D**): Levels of IFN-γ in whole brain lysates were measured using ELISA. Significant increase in pro-inflammatory cytokine levels in the brain up to 14 dpi for IL-6 and IL-1β and 30 dpi for IFN-γ and TNF-α compared to mock-infected mice. *n* = 4–8 mice per group. Statistical Analysis: One-way ANOVA (* *p* < 0.05; ** *p* < 0.01; *** *p* < 0.001; **** *p* < 0.0001 reflects the levels of statistical significance between groups).

Due to the presence of infectious SARS-CoV-2 in the brain post infection and previous studies showing elevated cytokine levels after SARS-CoV-2 infection, we assessed the levels of important pro-inflammatory cytokines in the brains of infected mice compared to mock. Notably, these inflammatory cytokines are linked with multiple neurodegenerative diseases, including Alzheimer’s disease. We observed a significant increase in the levels of IL-6, IL-1β, TNF-α and IFN-γ at 6 dpi compared to mock (Figure 3A–D). Interestingly, the high levels of TNF-α and IFN-γ were maintained at 14 dpi and 30 dpi, long after the virus had been cleared from the CNS. The levels of IL-6 and IL-1β dropped from their peak at 6 dpi by 14 dpi and 30 dpi; however, they were still higher than mock-infected mice. This chronic elevation of pro-inflammatory cytokines in the brain could induce several neurodegenerative pathways in the long term.

### 3.4. SARS-CoV-2 Infection Induces BBB Disruption

**Figure 4:**
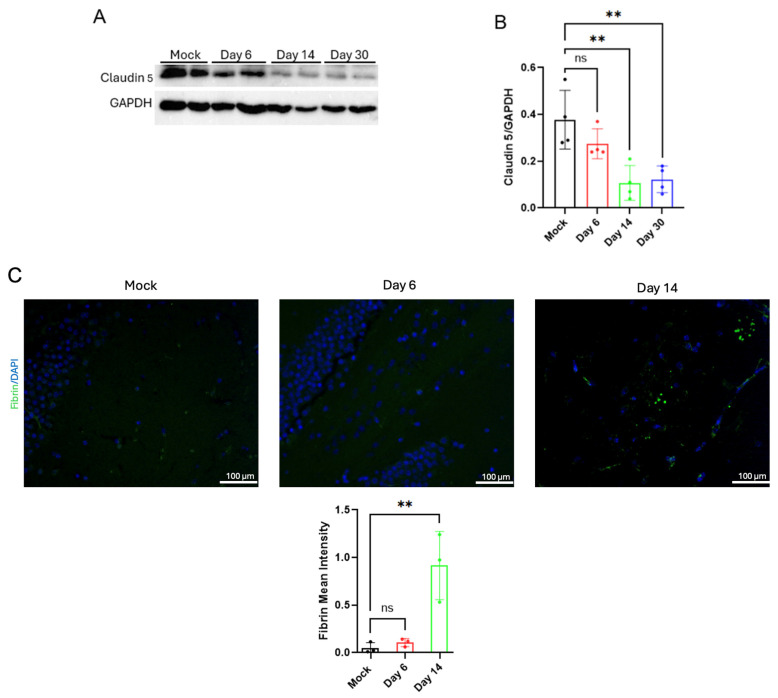
Decreased claudin-5 level and fibrin deposition in the brain of SARS-CoV-2-infected mice. (**A**): Western blot of whole brain lysates shows significantly reduced levels of claudin 5 in the brain at 14 dpi and 30 dpi. (**B**): Quantification of claudin-5 Western blot signals shows a significant reduction in the level of claudin-5 in infected mice at 14 dpi and 30 dpi vs. mock-infected mice. No significant difference between 6 dpi and mock. *n* = 4 mice per group. (**C**): Immunofluorescence assay for fibrin in brain tissues of infected and mock-infected mice (Magnification: 400×). Fibrin deposition was observed in the brains of infected mice at 14 and 30 dpi, showing BBB disruption at these time points. Arrows point at fibrin deposits fluorescent signals in the brain post infection. Statistical Analysis: One-way ANOVA (ns = no significance, ** *p* < 0.01; reflect the levels of statistical significance between groups).

Given the reduced number of pericytes in the brain post SARS-CoV-2 infection and the role of pericytes in BBB maintenance, we assessed claudin 5 levels and fibrin deposition in the brain. Claudin 5 is a tight junction protein that plays a crucial role in maintaining the integrity of the BBB, thus preventing the passage of harmful substances and microbes from the bloodstream into the brain. BBB breakdown is linked to neuroinflammation and neurodegeneration in conditions like AD and traumatic brain injury (TBI), leading to impaired cognitive function. We observed a significant decrease in Claudin-5 levels at 14 dpi and 30 dpi relative to mock (Figure 4A,B). In addition, fibrin was deposited in the brain of infected mice by 14 dpi but not in mock or 6 dpi mice. Interestingly, fibrin deposition at 14 dpi coincides with the timepoint when pericyte levels/numbers decrease in the brain (Figure 4C). These data suggest that SARS-CoV-2 infection induces BBB disruption and may potentiate fibrin-induced perivascular inflammation [26].

### 3.5. SARS-CoV-2 Infection Induces APP and Aβ1–42 Overexpression and Neuronal Death

**Figure 5:**
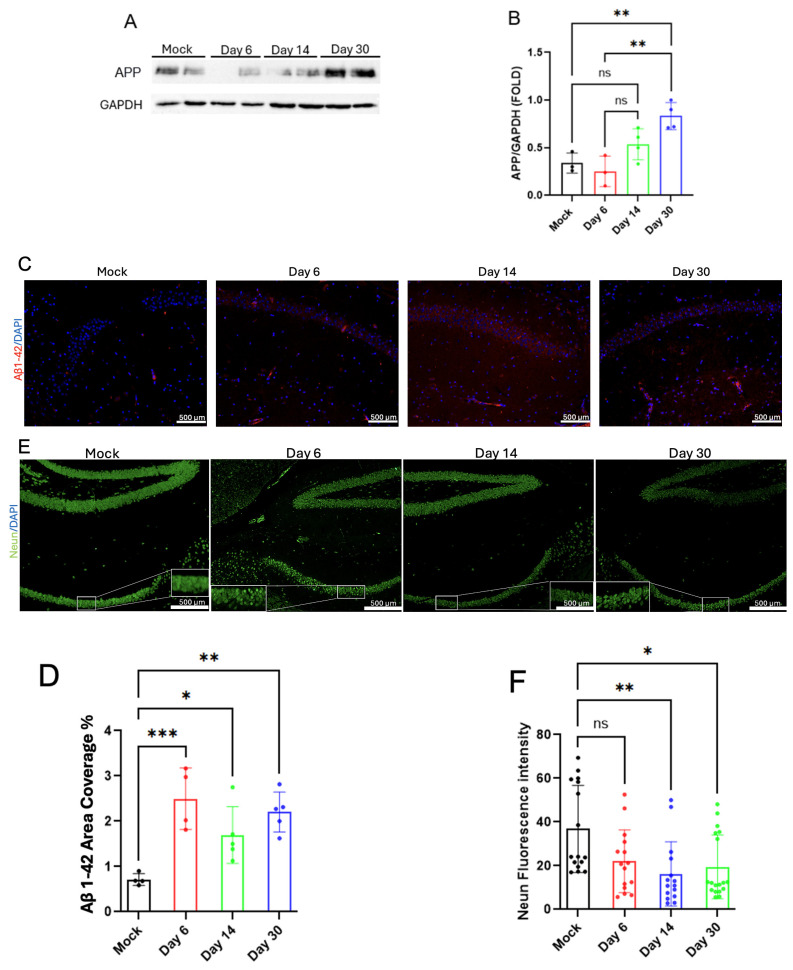
Increased APP expression and Aβ1–42 deposits, and decreased neuronal density in the brain of K18 mice post infection. (**A**,**B**): Western blot for amyloid beta precursor protein in the brain of infected and mock-infected mice. Blot shows a significantly increased level of amyloid beta in the brain at 30 dpi. *n* = 3–4 mice per group. (**C**): Immunofluorescence assay for Aβ1–42 following intranasal inoculation of SARS-CoV-2 in K18 mice. Arrows point at Aβ1–42 fluorescent signals in the brain (Magnification: 200×). (**D**): Quantification of amyloid beta 1–42 signals between the different groups. *n* = 4–5 mice per group. (**E**): Immunofluorescence assay for NeuN following intranasal inoculation of SARS-CoV-2 in K18 mice (Bar = 500 μm) (Magnification: 200×). (**F**): Quantification of NeuN signal per (20×) field between the different groups (16–18 fields per group). Statistical Analysis: One-way ANOVA (ns = no significance, * *p* < 0.05; ** *p* < 0.01; *** *p* < 0.001 reflect the levels of statistical significance between groups).

There is increasing evidence of the role of infectious diseases in AD. Aβ peptides exhibit antimicrobial characteristics and are likely to protect the brain against microbial infection. These peptides inhibit the growth of both Gram-negative and Gram-positive bacteria in vitro, and prevent herpesvirus entry into cells by rapidly seeding and binding to the viral surface glycoprotein [76,77,78]. In support of that, the Alzheimer’s disease mouse model (5XFAD) that overproduces Aβ survives longer and shows less disease severity when infected with HSV (intracranially) or Salmonella, to induce bacterial encephalomyelitis, compared to WT and APP knockout mice. APP KO mice showed the highest rate of mortality, and increased Aβ deposition in areas of the brain parenchyma that are infected [76,79]. This suggests that infection of the brain could initiate the aggregation of Aβ as a defense mechanism, but the deposits could serve as templates for further amyloid deposition. Consequently, we investigated the impact of SARS-CoV-2 infection on the level of Aβ in the brains of infected mice compared to mock. We observed a significant increase in amyloid-beta precursor protein expression in the brains of infected mice at 14 and 30 dpi by Western blot (Figure 5A,B). In addition, there was a significant increase in Aβ1–42 deposition in the brain from 6 dpi up to 30 dpi (Figure 5C,D). Decreased neuronal density in the hippocampal region is a major feature of AD. Thus, we examined the density of neurons in the hippocampal region of infected mice. Neuronal nuclei (NeuN) protein, also known as RBFOX3, is concentrated in the neuronal nucleus and is widely used as a specific marker for mature neurons. Interestingly, we observed a significant decrease in neuronal density in the hippocampus of infected mice at 14 dpi and 30 dpi relative to mock-infected mice by immunofluorescence using anti-NeuN antibody (Figure 5E,F). These findings indicate a higher risk of developing AD/other dementias in SARS-CoV-2 infected mice compared to mock.

### 3.6. SARS-CoV-2 Modulates the Expression of AD-Associated Genes in the Brains of Infected Mice

**Figure 6:**
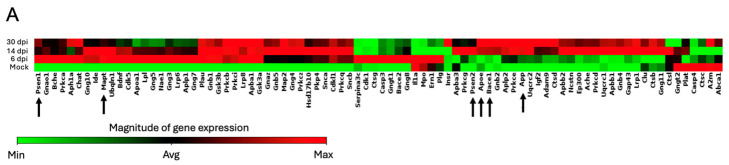

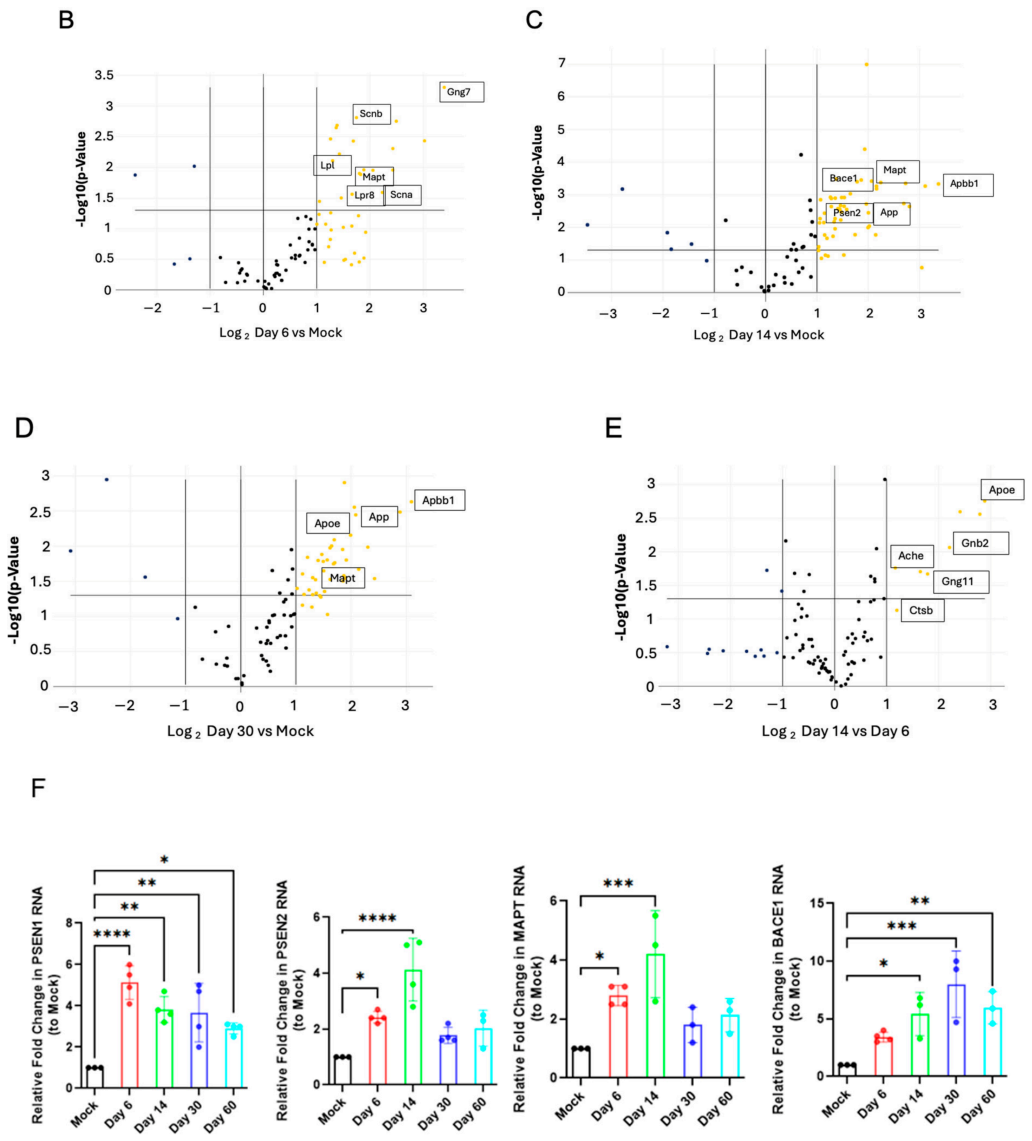
The transcriptional signature of AD-associated genes in the brains of infected mice. (**A**): Hierarchical clustering of genes illustrating changes in gene expression compared between infected mice and sham mice (*n* = 3 mice/group). Of the 84 genes studied, 19, 44, and 33 genes were significantly upregulated at 6 dpi, 14 dpi, and 30 dpi, respectively, compared to mock-challenged mice. (**B**): Volcano plots of differentially expressed genes (DEGs > 2.0-fold change, *p* < 0.05) between infected 14 dpi mice relative to mock-challenged mice. (**C**): Volcano plots of differentially expressed genes (DEGs > 2.0-fold change, *p* < 0.05) between infected 14 dpi mice relative to mock-challenged mice. (**D**): Volcano plots of differentially expressed genes (DEGs > 2.0-fold change, *p* < 0.05) between infected 30 dpi mice relative to mock-challenged mice. (**E**): Volcano plots of differentially expressed genes (DEGs > 2.0-fold change, *p* < 0.05) between infected 14 dpi mice relative to 6 dpi. Yellow dots represent upregulated genes. (**F**): RT-qPCR validation of some significantly upregulated DEGs in the brain of infected mice compared to mock. Statistical Analysis: One-way ANOVA (* *p* < 0.05; ** *p* < 0.01; *** *p* < 0.001; **** *p* < 0.0001 reflect the levels of statistical significance between groups).

Given the increase in Aβ in the brains of infected mice, neuroinflammation, BBB disruption, and other neuropathologies observed post-SARS-CoV-2 infection, we assessed the changes in the expression of AD-associated genes post-infection in our mouse model. Using a microarray-based system, we studied the transcriptional changes in 84 genes that play different roles in the development and progression of AD in the brains of mock-infected mice compared to infected mice at different timepoints. We observed 19, 44, and 33 significantly upregulated genes at 6 dpi, 14 dpi, and 30 dpi, respectively, compared to mock (Figure 6A–E). These differentially expressed genes (DEGs) are considered significant when there is ≥2.0-fold change (*p* < 0.05).

The 19 DEGs at 6 dpi include MAPT, the gene that encodes tau, a major hallmark of AD. Further analysis shows a significant increase in genes like apolipoprotein 1 (Apoa1), lpr-8, Snc-α, and Snc-β, all of which play key roles in AD development. Apoa1 facilitates the spread of alpha-synuclein and tau, thus inducing neurotoxicity in many types of dementia and Parkinson’s disease [80,81]. Alpha-synuclein forms fibrils that are found in Lewy bodies in the brains of patients with dementia and exacerbate tau-mediated neurotoxicity [82,83].

The 44 DEGs at 14 dpi and the 33 DEGs at 30 dpi include MAPT, APP, APOE, and BACE1. Amyloid precursor protein (APP) overexpression is a key factor in the development of AD. Overexpression of APP in mice induces neuronal death and cognitive decline [84]. APOE4 is the strongest genetic factor for AD. In addition, APOE overexpression, regardless of variant, induces neuropathology. APOE overexpression in the frontal cortex of mice induces anxiety-like behavior and learning deficits [85]. Neuronal APOE drives neuroinflammation, tau accumulation, and neuronal death through upregulating the MHC-I expression [86]. BACE1 cleaves APP into soluble APPβ and C99 in the pathogenic amyloidogenic pathway of APP processing in the brain. The C99 produced by this cleavage can be further cleaved by γ-secretase to produce pathogenic Aβ, and this is a rate-limiting step in the production of Aβ1–42 in the brain [87,88]. In addition, PSEN1, one of the two proteins that make up the catalytic domain of γ-secretase, was overexpressed in 14 dpi mice brains compared to mock, thus increasing the probability of Aβ production. There were 12 and 11 DEGs at 14 dpi and 30 dpi, respectively, compared to 6 dpi. These genes include Apbb1, APOE, APP, BACE1, and Ctsb. Furthermore, we validated some essential DEGs using RT-qPCR and observed significant differences in the expression of BACE1, PSEN1, PSEN2, and MAPT (Figure 6F).

### 3.7. Signaling Pathway Analysis

**Figure 7:**
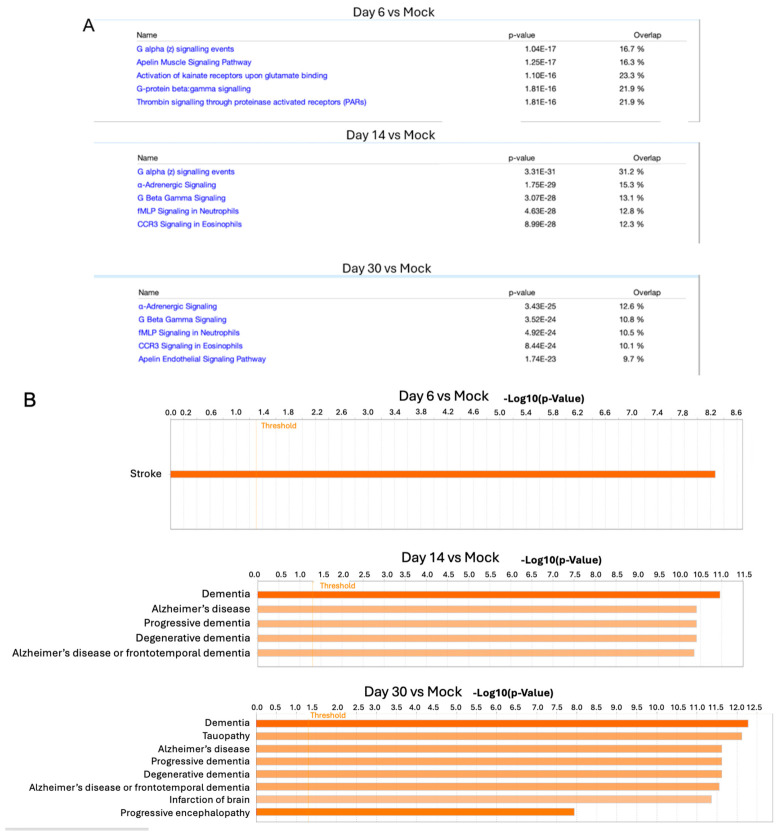
Top canonical pathways identified from Ingenuity Pathway Analysis of DEGs in the brains of SARS-CoV-2-infected mice. Top canonical pathways identified and ML disease prediction by Ingenuity Pathway Analysis (IPA). (**A**) Top 5 pathways identified in infected mice compared to mock, ranked according to their –log (*p*-value). (**B**): ML disease prediction by IPA shows increased risk of chronic neurodegenerative disease, such as AD and Tauopathy, in infected mice by Day 14 and Day 30. Color intensity denotes the magnitude of fold change in gene expression.

The DEGs obtained from transcriptomic analysis were uploaded to Ingenuity Pathway Analysis (IPA) to identify dysregulated signaling pathways in the brains of infected mice compared with mock-infected mice. The criteria for DEGs were fold change > 2 and *p*-value < 0.01. Canonical pathways were derived and ranked by the ratio of DEGs to the number of genes mapped to each pathway (Figure 7A). Analysis shows upregulation of ‘G beta-gamma (Gβγ) signaling’ in the brains of all the groups of infected mice compared to mock. Gβγ enhances Aβ toxicity through the formation of the APP/Go Gβγ complex, thus inducing neuronal death and dysfunction [89,90]. In addition, ‘G-alpha z (Gα_z_) signaling’ was upregulated at 6 and 14 dpi compared to mock. Gα_z_ signaling regulates neurotransmitter release in the brain and behavioral response by coupling neurotransmitter receptors to N-type Ca^2+^ channels when slightly overexpressed in neurons [91]. ‘Thrombin signaling through proteinase-activated receptors (PARs)’, dysregulated at 6 dpi, is a double-edged sword as it is neuroprotective at low concentrations in the brain. However, it induces neuroinflammation, BBB disruption, and other neuropathological complications at high concentrations in the brain [92,93,94]. The second top canonical pathway activated at 6 dpi is the ‘apelin muscle signaling pathway’, a neuroprotective pathway that helps reduce inflammation and apoptosis in the brain [95,96], showing an attempt to maintain brain homeostasis at this timepoint. In addition to Gβγ signaling, α-adrenergic signaling was upregulated at 14 and 30 dpi compared to mock. α-adrenergic signaling plays a key role in memory formation and synaptic plasticity. However, upregulation of the signaling pathway induces cognitive impairment, reduces cerebral blood flow, and increases anxiety [97,98,99]. Based on the DEGs analyzed with IPA core analysis, IPA disease machine learning predicted stroke at 6 dpi compared to mock (Figure 7B). This supports previous studies that show that COVID-19 leads to a higher risk of stroke, especially during the acute phase of infection [94,100,101]. Studies show that COVID-19 induces stroke through fibrinogen-mediated hypercoagulability, cytokine storm, and damage to blood vessels [101,102,103], and stroke increases mortality risk in COVID-19 patients [104]. Furthermore, dementia, tauopathy, AD, and infarction of the brain were predicted at 14 dpi compared to mock. For 30 dpi, AD and dementia were predicted based on the upregulated genes such as APP, MAPT, PSEN, and BACE1 from the RT^2^ PCR microarray analysis.

The top 3 significantly upregulated pathways at 14 dpi and 30 dpi compared to 6 dpi are ‘Activation of kainate receptors upon glutamate binding’, ‘Gβγ signaling’, and ‘thrombin signaling through proteinase-activated receptors (PARs)’. Kainate receptors (KARs) overactivation induces Ca^2+^ influx in neurons, neuronal excitotoxity, and neuronal death (apoptosis), especially in the hippocampal region of the brain [105,106,107]. Overactivation of KARs has been implicated in neurological conditions like ischemia, Huntington’s disease, and AD. However, no disease was predicted between 14 dpi and 30 dpi compared to 6 dpi.

## 4. Discussion

Although COVID-19 is a respiratory disease, extrapulmonary complications are common, affecting up to 33% of patients during and after the acute phase of infection [108,109,110]. Previous reports have linked these complications to direct viral neuroinvasion [111,112]. However, the mechanism involved in the pathological changes in the brain post SARS-CoV-2 infection, especially in the context of long-COVID, is not fully understood. In this study, we revealed that SARS-CoV-2 directly infects the brain, induces neuroinflammation, pericyte death, and multiple perivascular changes in the brain. Our main novel finding in this report is the virus-induced depletion of brain pericytes, which may occur via a combination of direct virus infection and indirect inflammatory cell death of pericytes. Loss of pericyte function induces BBB disruption, thus potentiating neuropathological complications downstream of BBB breakdown. These neuropathological and neurovascular manifestations persisted even after the virus had been fully cleared from the brain. This discovery highlights the potential of the virus to induce chronic neuropathology and, by extension, neurodegenerative diseases such as AD and related dementias that relate to BBB and pericyte dysfunction.

Brain pericytes regulate the blood–brain barrier, modulate inflammation, and clear toxic materials such as amyloid beta from the brain [28]. All these functions help maintain homeostasis in the brain, and brain pericyte death and dysfunction contribute to several neurological diseases. To elucidate the effect of SARS-CoV-2 infection on the abundance of brain pericytes, we analyzed the level of CD13 in the brains of infected mice compared to uninfected mice. First, we observed no significant difference between mock-infected mice and 6 dpi mice. However, there was a drastic and significant reduction in pericyte numbers in the brain by 14 dpi, and as expected, pericyte numbers remained decreased at least until 30 dpi. In addition, we obtained evidence of cell death in the brain of infected mice via TUNEL staining colocalized with CD13, thus suggesting that pericytes died by an apoptotic pathway. There was also an inverse correlation between pericyte levels and FLI-1 at the observed timepoints, supporting previous reports of FLI-1 overexpression during pericyte death and dysfunction [113,114]. Interestingly, pro-inflammatory cytokines associated with vascular inflammation, including TNF-α, induce pericyte migration and apoptotic cell death at high concentrations [115,116,117], suggesting a possible pathway besides direct infection of ACE2^+^ pericytes by which SARS-CoV-2 induces pericyte cell death. In this alternative pathway, the presence of SARS-CoV-2 in the brain could drive the initial inflammation, and the induction of fibrin-mediated neuroinflammation could play an important role in perpetuating neuroinflammation and pericyte cell death after virus clearance. However, the exact pathways for virus-induced cell death of pericytes will require further investigation in this model.

Cytokine storm is one of the hallmarks of SARS-CoV-2 infection, and it has been linked not only to the development of acute respiratory disorder but also to the extrapulmonary organ damage [118]. This highlights the crucial roles inflammatory cytokines play in COVID-19 pathogenesis. Moreover, neuroinflammation plays a cardinal role in the development of multiple chronic neurological diseases. Considering this, we assessed the levels of pro-inflammatory cytokines (IL-6, IFN-γ, IL-1β, and TNF-α) in the brains of infected mice compared to mock. As expected, there was a drastic increase in the level of all pro-inflammatory cytokines in the brain by 6 dpi. The levels of IL-6 and IL-1β dropped from the 6 dpi peak by 14 and 30 dpi to slightly above mock. Interestingly, the high levels of TNF-α and IFN-γ are maintained up to 14 and 30 dpi. This is notable because TNF-α and IFN-γ have been shown to synergistically induce PANoptosis, a highly inflammatory cell death pathway, and it has been reported that these two cytokines are elevated in COVID-19 patients [119,120]. Furthermore, TNF-α has both pros and cons in the brain as it helps in the formation of synaptic connections in the brain at homeostatic levels. However, it induces neuronal death due to excessive stimulation from excitatory transmitters and BBB disruption at pathological levels in the brain [121]. Further studies are needed to elucidate how this chronic neuroinflammation is maintained, but we speculate that TNF-α and IFN-γ induced PANoptosis may explain the vicious cycle of neuroinflammation. In this study, there is a limitation because we histologically examined inflammatory cells but did not identify the specific types of inflammatory/immune cells in the brains of SARS-CoV-2-infected mice. Inflammatory cell influx refers to the recruitment of peripheral immune cells (e.g., monocytes, neutrophils, and lymphocytes) into the brain following disruption of the blood–brain barrier or chemokine-mediated trafficking. In contrast, inflammatory cell expansion refers to the increase in resident immune cell populations, primarily through local proliferation and/or activation of microglia and other brain-resident immune cells. To distinguish these mechanisms experimentally, new experiments would be designed to separately evaluate peripheral immune cell infiltration and resident immune cell expansion in our future studies. Peripheral immune cell influx will be quantified by flow cytometry and immunohistochemistry using markers that distinguish infiltrating leukocytes from resident microglia (e.g., CD45^high^ CD11b^+^ versus CD45^int^ CD11b^+^, together with Ly6C, Ly6G, and CCR2 where appropriate). Resident microglial expansion will be assessed by proliferation markers (Ki67 or BrdU/EdU incorporation) combined with microglial markers (TMEM119 and P2RY12) and by quantification of cell density.

Systemic inflammation and BBB disruption have been observed in COVID-19 patients, particularly in those with long COVID-associated neurological impairment [15,122,123]. BBB breakdown occurs in the hippocampus very early in the development of cognitive dysfunction and has been suggested as an early marker of chronic neurological diseases [16,124]. BBB disruption has been observed both in AD patients and AD mouse models [13,17]. Herein, we assessed BBB disruption in the brain post SARS-CoV-2 infection. First, we observed a decrease in claudin-5 levels in the brains of infected mice at 14 dpi and 30 dpi compared to mock. Claudin-5 helps in maintaining the integrity of the BBB, thereby preventing the passage of harmful substances and microbes from the bloodstream into the brain. Claudin-5 is primarily produced by the epithelial cells with the help of pericytes through the secretion of glial cell line-derived neurotrophic factor (GNDF) by pericytes [125,126]. Thus, it is important to note that the time point at which we observed a significant decrease in claudin-5 levels coincided with the pericyte loss. Subsequently, we assessed fibrin deposition in the brain and observed fibrin deposition in the hippocampal region of 14 dpi mice, but not in mock or 6 dpi mice. Fibrin drives neuroinflammation by activating perivascular macrophages and parenchymal microglia, inducing cytokine production and oxidative injury [127], and it has been identified as a potential culprit in COVID-19-associated neuroinflammation [128]. Given our findings of fibrin deposition post-infection, we speculate that fibrin deposition in the brain may occur through virus-induced fibrinogen-mediated inflammation of the BBB, exacerbated by pericyte loss via inflammation and/or direct infection by SARS-CoV-2.

Multiple epidemiological studies show a link between COVID-19 and AD [19,20,22]. However, further behavioral studies are needed to confirm the potential role of SARS-CoV-2 in AD in our model. Herein, we showed that SARS-CoV-2 infection induces amyloid precursor protein overexpression, increases Aβ1–42 deposits in the brain, and reduces neuronal density in the hippocampus. All these findings underpin epidemiological studies linking COVID-19 to AD and suggest that further research is needed to fully elucidate the mechanisms involved. Aβ elicits anti-microbial properties, thus we speculate that direct or indirect effects of SARS-CoV-2 infection of the brain initiate pericyte loss and Aβ seeding for eventual lesion development. As a possible antiviral role for Aβ, our data could explain the significant increase in Aβ1–42 observed by 6 dpi in the absence of a significant increase in APP production. The increased Aβ1–42 might be a response to viral presence in the brain, leading to more efficient cleaving of APP to produce Aβ1–42 to aid virus clearance. However, this unregulated Aβ aggregation becomes neuropathological, inducing a positive feedback mechanism, especially due to the presence of other neuropathological conditions like BBB disruption, pericyte dysfunction, and chronic neuroinflammation. The long-term persistence (30 dpi) of increased Aβ AND APP production in the brain, coupled with neuronal death, indicates that these mice are at high risk of developing ADRD.

Furthermore, we observed a significant increase in multiple Alzheimer’s disease-related genes. Notably, MAPT, the gene encoding tau, increased from 6 dpi through 30 dpi, suggesting tauopathy, an important component of AD pathology at all time points. In addition, APP, BACE1, and PSEN1 were significantly increased at 14 dpi through 30 dpi. PSEN2 was also slightly increased at 14 dpi and 30 dpi, although the increases were not statistically significant. All these genes play crucial roles in the Aβ processing. A significant increase in APP implies a greater chance of Aβ production due to a higher substrate for the BACE1 enzyme to act on. In addition, APP overexpression in the brain has been shown to lead to neuronal death and cognitive deficit even before the accumulation of Aβ plaques [84,129]. The BACE1 enzyme cleaves APP at the N-terminus of the Aβ sequence. The products of APP cleavage by BACE1 are soluble APPb (sAPPb, which is released extracellularly) and a membrane-bound C99 fragment. This process is followed by the intramembranous cleavage of the membrane-bound C99 fragment at the epsilon (e) site by γ-secretase to produce Aβ-48 and APP intracellular domain (AICD), which is released into the intracellular space. The γ-secretase continues to sequentially cleave Aβ-48, producing shorter peptides in the process until the amyloid beta (usually 38–43 amino acids long) is released extracellularly from the complex. γ-secretase’s catalytic domain comprises PSEN1 and PSEN2, which were also increased at 14 dpi and 30 dpi.

Lastly, ingenuity pathway analysis demonstrates the activation of multiple pathways in the brains of infected mice, thus disrupting brain homeostasis. Overactivation of signaling pathways such as Gβγ signaling, α-adrenergic signaling, and thrombin signaling pathways are linked to multiple neurodegenerative disorders and neuronal death. Inhibiting Gβγ with Gallien, a selective pharmacological inhibitor, inhibited Aβ-induced neuronal death and improved memory impairment in 3xTg-AD mice when applied intrahippocampally. Interestingly, one of the top 2 upregulated pathways at 6 dpi is an anti-inflammatory and anti-apoptotic pathway called ‘apelin muscle signaling pathway’, showing an attempt to reestablish homeostasis and reduce inflammation at this timepoint. In addition, IPA machine learning (ML) disease prediction analysis predicted AD, tauopathies, and dementia at 14 dpi and 30 dpi, providing strong evidence to support epidemiological studies that show an increase in the probability of developing dementia post COVID-19.

It should be noted that the K18 mouse model has been an important tool in COVID-19 research due to its high sensitivity to the virus compared to other mouse models. However, the pattern of hACE2 expression in this mouse does not fully match the physiological expression pattern [130]. This may explain the failure to observe co-localization of CD13 and SARS-CoV-2 N-protein signals in this study, as observed in postmortem human brain samples [33] and our hACE-2 knock-in (KI) model (Supplementary Figure S3). Importantly, this observation suggests that SARS-CoV-2-induced inflammation could drive pericyte death in the absence of direct viral infection.

**Figure 8:**
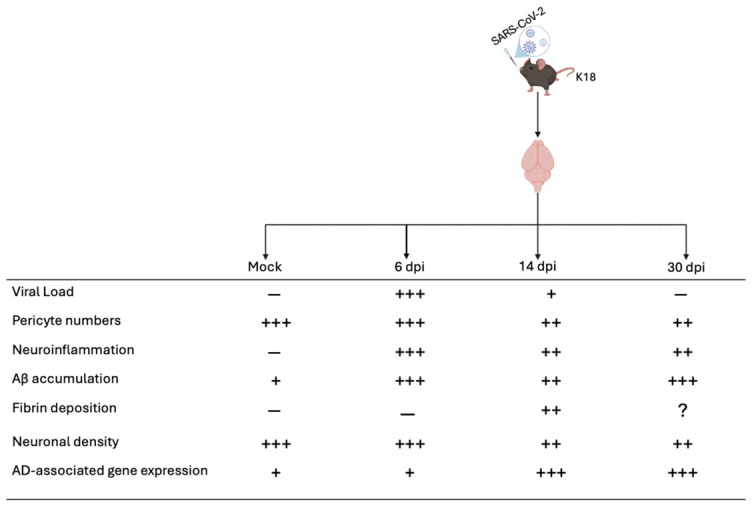
A summary of COVID-19-induced neuropathological changes in the K18 mouse model. SARS-CoV-2 infection induces dynamic neuropathological changes in neuroinflammation, BBB disruption, APP and Aβ deposition, reduced hippocampus neuron density, and increased AD-related gene expression and activation of signaling pathways in the brain. Legend: (+++ = high level; ++ = medium; + = low; − = not detected; ? = not tested).

In summary, we showed multiple neuropathological pathways as shown in Figure 8: (1) pericyte death, (2) BBB disruption and fibrin deposition, (3) Aβ accumulation, (4) chronic inflammation, (5) neuronal death, and (6) an increased expression in AD-associated genes like MAPT, PSEN1 and APP, as well as activity of several AD-relevant pathways like Gβγ signaling, KARs activation by glutamate, and thrombin signaling that are known to contribute to AD development in SARS-CoV-2 infected mice.

## Data Availability

The original contributions presented in this study are included in the article/Supplementary Material. Further inquiries can be directed to the corresponding author.

## Supplementary Materials

The following supporting information can be downloaded at: https://www.mdpi.com/article/10.3390/v18070783/s1, Figure S1: Survival curve post SARS-CoV-2 infection in K18 mice; Figure S2: Co-staining with CD13 and N-protein antibodies in the brain of infected K18 mice; Figure S3: Co-staining with CD13 and N-protein antibodies in the brain of infected Ki mice. Arrows point to areas of co-localization.
