# Supplementary material for: Neuroinflammation, Pericyte Dysfunction, and Alzheimer’s Disease-Associated Gene Expression and Pathway Activation in the Brain of SARS-CoV-2-Infected Mice
Source: Viruses. 2026 Jul 17;18(7):783. doi: 10.3390/v18070783 (PMC13431636; doi:10.3390/v18070783)
Supplement: Supplementary file 1 [file viruses-18-00783-s001.zip › viruses-4291573-supplementary.pdf]

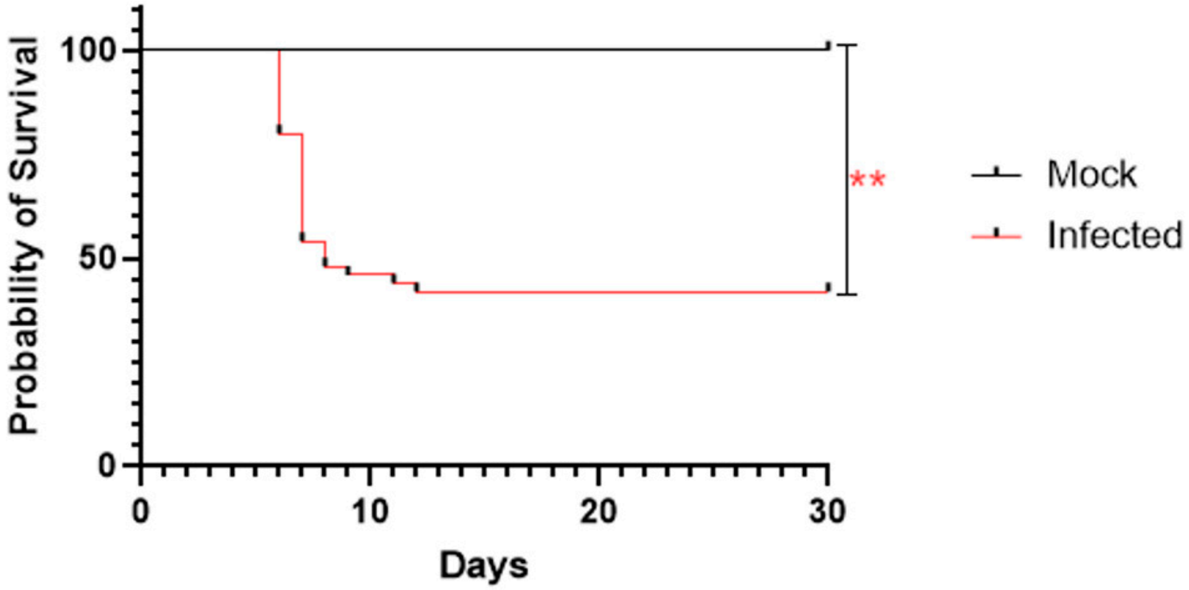


**Supplementary Figure S1: Survival curve post SARS-CoV-2 infection in K18 mice.** Survival curve post-SARS-CoV-2 infection shows approximately 50% survival rate in K18 mice.

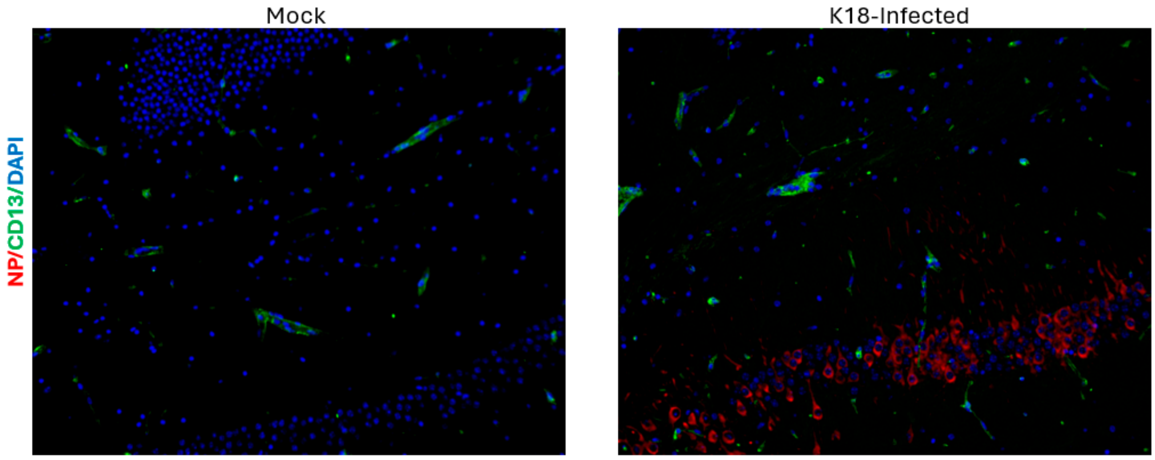


**Supplementary Figure S2. Co-staining with CD13 and N-protein antibodies in the brain of infected K18 mice.** Co-staining with CD13 and N-protein antibodies shows no co-localization of CD13 and SARS-CoV-2 N-protein fluorescent signals in the brain of infected K18 mice.

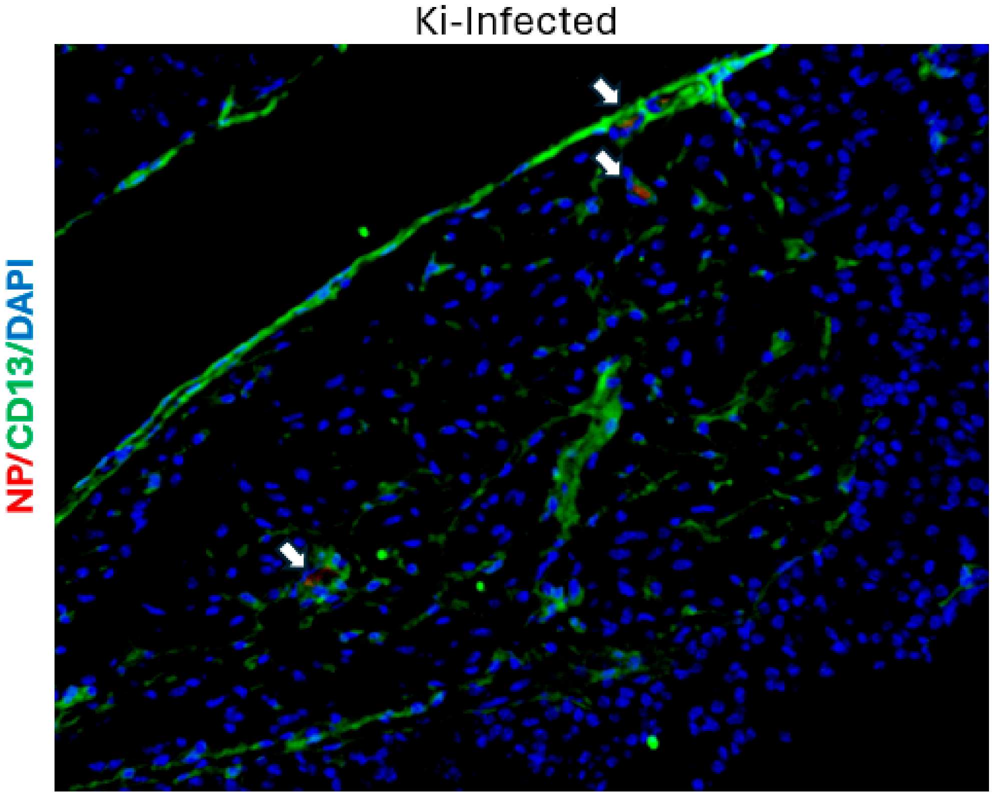


**Supplementary Figure S3. Co-staining with CD13 and N-protein antibodies in the brain of infected Ki mice**. Co-staining with CD13 and N-protein antibodies shows co-localization of CD13 and SARS-CoV-2 N-protein fluorescent signals in the brain of infected Ki mice. Arrows point to areas of co-localization.
